# Internet-delivered acceptance-based CBT for frequent migraine in routine care as add-on to medical treatment 12-month results: A randomized controlled trial^☆^

**DOI:** 10.1016/j.invent.2026.101022

**Published:** 2026-11-05

**Authors:** Marie Persson, Anna Sundholm, Bledar Daka, Nashmil Ariai, Sandra Weineland

**Affiliations:** a Family Medicine, School of Public Health and Community Medicine, Sahlgrenska Academy, University of Gothenburg, Gothenburg, Sweden; b Research and Development, Primary Health Care Region Västra Götaland, Sweden; c Department of Neuroscience, Karolinska Institutet, Stockholm, Sweden; d Department of Neurology, Karolinska University Hospital, Stockholm, Sweden; e Department of Psychology, University of Gothenburg, Gothenburg, Sweden

**Keywords:** Migraine, Randomized controlled trial, Internet-based treatment, ICBT, Acceptance and commitment therapy, Primary care

## Abstract

**Background:**

Frequent episodic and chronic migraine are associated with disability, psychological distress, and reduced quality of life. This study evaluated the long-term effects of I AM, an individually tailored, therapist-guided, acceptance-based, Internet-delivered cognitive behavioral therapy (ICBT) programme combined with treatment as usual (TAU) in Swedish primary care.

**Objective:**

The aim was to evaluate the effects of acceptance-based Internet-delivered cognitive behavioral therapy + TAU compared with TAU alone on migraine frequency, pain intensity, migraine-related disability, and mental health and well-being.

**Methods:**

In this randomized controlled study, 154 participants with frequent episodic or chronic migraine were randomized to *I AM* + TAU or TAU alone. Outcomes were assessed at baseline, post-treatment, and at 6- and 12-month follow-ups. Intervention effects were evaluated using time × condition interactions, with baseline as the reference timepoint.

**Results:**

For migraine-related disability (HIT-6), the time × condition interaction was significant at 12 months (β = −3.406, 95% CI [−5.970, −0.841], *p* = .010), indicating a greater reduction from baseline in the *I AM* + TAU group compared with TAU alone. For mental health and well-being (MHC-SF), significant time × condition interactions were observed at post-treatment (β = 5.472, 95% CI [0.071, 10.873], *p* = .047) and 12 months (β = 5.563, 95% CI [0.176, 10.950], *p* = .043), indicating greater improvements in the *I AM* + TAU group. The interaction at 6 months was not significant for either HIT-6 or MHC-SF. No significant time × condition interactions were observed for number of monthly migraine days or pain intensity at post-treatment, 6 months, or 12 months.

**Conclusions:**

*I AM* combined with TAU resulted in greater reduction in migraine-related disability at 12 months and in mental health and well-being at post-treatment and 12 months compared with TAU alone. No differences between the groups were observed on number of monthly migraine days or pain intensity. These findings suggest that acceptance-based ICBT may provide benefits primarily in reducing the functional burden of migraine and supporting psychological well-being rather than in reducing migraine frequency or pain intensity.

## Highlights

• *I AM* reduced daily migraine burden and improved adaptation to living with migraine.
• *I AM* participants showed greater improvements in mental health and well-being.
• Results highlight integrated migraine care where psychological and medical work together.
• *I AM* is feasible and accepted as internet care for frequent episodic and chronic migraine in primary care.

## 1 Introduction

Migraine is a common and disabling neurological disorder characterized by recurrent headache attacks that are typically moderate to severe in intensity and may be unilateral and pulsating, aggravated by routine physical activity, and accompanied by symptoms such as nausea and sensitivity to light and sound (International Headache Society [IHS], 2018). Migraine can substantially affect quality of life, everyday functioning, and work capacity, with considerable societal costs (Martelletti et al., 2018; Hjalte et al., 2019). Episodic migraine is characterized by migraine occurring on fewer than 15 headache days per month, whereas chronic migraine is defined as headache occurring on ≥15 days per month for more than three months, with migraine features present on at least 8 days per month (IHS, 2018). Globally, migraine affects approximately 14% of the population, while chronic migraine affects approximately 2% (Stovner et al., 2018). Individuals with frequent episodic or chronic migraine may experience substantial disease burden both during and between attacks (Burch et al., 2019; Whitaker et al., 2024; Matharu et al., 2025).

Migraine treatment in primary care commonly includes acute and preventive pharmacological treatment. However, behavioral and psychological interventions may provide additional benefits when integrated with medical care. Randomized controlled trials have shown that combining behavioral interventions with pharmacological treatment can improve outcomes such as migraine frequency, disability, and functioning (Holroyd et al., 2010; Grazzi et al., 2023). The course and burden of migraine are influenced by interacting biological, behavioral, psychological, and social factors, including disease characteristics, comorbidities, lifestyle factors, and pain-management strategies (Lipton et al., 2023). A biopsychosocial perspective may therefore complement medical management by addressing behavioral, psychological, and functional consequences of migraine (Rosignoli et al., 2022). Rather than directly targeting the underlying neurological mechanisms of migraine, psychological interventions may help individuals develop more adaptive ways of responding to symptoms, distress, and the impact of migraine on everyday life. Psychological treatments for migraine vary considerably in both therapeutic content and delivery format (Lee et al., 2019; Stubberud et al., 2025).

Internet-delivered cognitive behavioral therapy (ICBT) offers one means of increasing access to psychological interventions and has been applied to chronic pain conditions, including migraine (Buhrman et al., 2016; Buhrman et al., 2023; Hedman-Lagerlöf et al., 2023). Internet-delivered interventions for migraine have incorporated components such as psychoeducation, stress management, self-management strategies, and acceptance-based approaches, with studies indicating potential benefits across clinical and functional outcomes (Bromberg et al., 2012; Kindelan-Calvo et al., 2014). Acceptance-based approaches may be particularly relevant in this context because they broaden the therapeutic focus beyond symptom control to include how individuals respond to and function in the presence of recurring symptoms (Polk and Smitherman, 2023).

Acceptance and Commitment Therapy (ACT) is a transdiagnostic behavioral and cognitive therapy that aims to increase psychological flexibility, broadly defined as the capacity to remain open to internal experiences while adapting behavior in ways that are consistent with personally meaningful values (Hayes et al., 2006). Rather than primarily seeking to eliminate unwanted thoughts, emotions, or physical sensations, ACT focuses on changing how individuals relate and respond to these experiences. Psychological flexibility is commonly conceptualized through six interrelated processes: acceptance, cognitive defusion, present-moment awareness, self-as-context, values clarification, and committed action (Hayes et al., 2006; Hayes et al., 2012) Together, these processes aim to support flexible and context-sensitive behavior rather than rigid patterns of experiential avoidance or symptom control.

This framework may be particularly relevant to migraine because the burden of the condition can extend beyond the migraine attack itself. The episodic and often unpredictable nature of migraine may contribute to anticipatory worry, heightened attention to bodily sensations, concerns about potential triggers, and changes in everyday activities. Migraine attacks may also disrupt work, family life, and other valued activities, creating demands to compensate for activities missed during an attack. Although attempts to predict, control, or avoid migraine-related experiences can be adaptive in some circumstances, rigid patterns of avoidance and symptom control may restrict behavioral repertoires and interfere with everyday functioning and valued activities (Martin and MacLeod, 2009; Varkey et al., 2013; Hubig et al., 2022). From an ACT perspective, treatment therefore aims to increase the individual's capacity to respond flexibly to pain-related sensations, thoughts, emotions, and uncertainty while supporting engagement in personally meaningful activities even when complete symptom control is not possible (Hayes et al., 2006; McCracken and Vowles, 2014).

Research in chronic pain provides support for the relevance of ACT and psychological flexibility in the management of persistent pain. Acceptance, values-based action, and related flexibility processes have been associated with functioning and psychological outcomes in chronic pain (McCracken and Vowles, 2014), and systematic reviews and meta-analyses of ACT for chronic pain have reported beneficial effects across outcomes including pain-related functioning, pain acceptance, and psychological flexibility (Trindade et al., 2021, Ma et al., 2023). ACT has also been delivered through guided Internet-based interventions, with studies indicating beneficial effects in chronic pain populations (Buhrman et al., 2013, Lin et al., 2017, Trindade et al., 2021). Evidence specifically concerning acceptance-based interventions for migraine is more limited. A meta-analysis of ACT and mindfulness-based interventions for adults with migraine found significant improvements in migraine-related disability, whereas effects on headache frequency and medication use were not statistically significant (Polk and Smitherman, 2023).

The *I AM* intervention was developed within this acceptance-based framework. ACT constitutes a central therapeutic component of *I AM* and is complemented by mindfulness- and compassion-oriented strategies according to individual needs. Mindfulness- and compassion-oriented strategies may provide complementary approaches within acceptance-based treatment. Mindfulness-based strategies emphasize present-moment, non-judgmental awareness of internal experiences and have been investigated specifically in individuals with migraine (Wells et al., 2021; Prohaska and Matthias, 2023). Qualitative research further suggests that mindfulness practice may influence migraine-related experiences through processes such as increased acceptance and self-compassion and reduced anticipatory anxiety and pain reactivity (Estave et al., 2023). Compassion-oriented strategies, in turn, may be particularly relevant for addressing self-criticism and supporting more adaptive self-care in the context of persistent pain (Malpus et al., 2023). Although the evidence for compassion-based approaches specifically in migraine remains limited, emerging research indicates that self-compassion may be relevant to psychological distress and disease-related experiences among individuals with migraine. Accordingly, mindfulness- and compassion-oriented strategies were incorporated in *I AM* as complementary components alongside the central acceptance-based framework. These components overlap with, and may support, broader processes related to psychological flexibility. *I AM* is individually tailored through the selection of treatment modules and adaptation of treatment pacing and therapist support. This is consistent with a process-based perspective in which therapeutic components can be selected according to psychological processes and difficulties relevant to the individual rather than relying exclusively on a fixed treatment protocol (Hayes and Hofmann, 2021). Thus, *I AM* combines acceptance-based principles with individually selected therapeutic components to address migraine-related symptoms, coping, functioning, and psychological well-being.

A pilot randomized controlled trial (*n* = 29) conducted in Swedish primary care evaluated the feasibility and acceptability of *I AM* as a therapist-guided, acceptance-based ICBT intervention for individuals with frequent episodic migraine (Persson et al., 2024). The pilot indicated that the digital format and treatment approach were feasible and generally acceptable, while also identifying aspects of the intervention that could be further developed. These findings informed the subsequent full-scale evaluation and refinement of *I AM*, including its individually tailored format.

The present randomized controlled trial evaluates *I AM* as an adjunct to treatment as usual (TAU) in Swedish primary care among individuals with frequent episodic or chronic migraine. The primary aim was to examine whether changes over time differed between participants receiving *I AM* in addition to TAU and those receiving TAU alone. Outcomes included migraine frequency, pain intensity, migraine-related disability, and mental health and well-being, assessed from baseline through post-treatment, 6-month follow-up, and 12-month follow-up.

## 2 Methods

### 2.1 Study design

This study employed a longitudinal randomized controlled trial (RCT) conducted within primary health care in Sweden. Outcomes were assessed at baseline, posttreatment (four or twenty weeks depending on the number of modules and module frequency chosen by the participant), 6 - and at 12-month follow-up, following CONSORT reporting guidelines (Schulz et al., 2010).

### 2.2 Procedure

Participants were recruited between January 2023 and March 2024 from healthcare centers in the Västra Götaland region of southwestern Sweden. Participants, diagnosed with episodic or chronic migraine, were recruited through a variety of channels. Advertisements were placed in both public and private healthcare centers, on social media platforms managed by the primary healthcare Research and Development (R&D) unit (such as LinkedIn and Facebook), in newspapers, and spread through the Region Västra Götaland's local Headache Association group. Staff at two primary care centers sent out letters with information about the study to patients who had visited the center for migraines within the last two years. Inclusion criteria required participants to be at least 18 years old, registered at a primary care center within the region, diagnosed with episodic or chronic migraine with three or more migraine days per month. Participants needed access to a computer, tablet, or smartphone with BankID and an internet connection. Exclusion criteria included individuals with active malignant diseases, as well as other rare headache disorders such as cluster headache, basilar migraine, vestibular migraine, and ocular migraine, or severe mental illness such as severe depression or anxiety, neuropsychiatric disorders, psychotic conditions, substance abuse, or personality disorders. However, individuals with moderate mental health issues who maintained overall functional stability were eligible. Furthermore, individuals unable to understand Swedish were excluded due to the language requirements of the intervention, which was available only in Swedish.

Eligible participants who expressed interest were invited for an initial assessment by a psychologist. In this assessment, participants were screened for inclusion and exclusion criteria and received oral and written study information. At the time of contacting the study, participants had already received a migraine diagnosis from a physician in primary care or a neurologist at a specialist clinic. Participants self-reported their diagnoses based on this information. Participants who did not have an established migraine diagnosis when they contacted the study were encouraged to consult their primary care physician for diagnostic evaluation. The psychologist who conducted the assessment interview with the participant collected data of headache symptoms, duration, estimated work capacity and information about the medications they use for their migraine condition. A structured interview was conducted using the Primary Care Evaluation of Mental Disorders (PRIME-MD) to assess potential comorbid psychological symptoms (Tamburrino et al., 2009). During the assessment interview, the psychologist and the participant selected which modules, treatment pace and degree of therapist support the participant needed.

During the assessment interview, both the psychologist and the participants were blinded to group allocation. Randomization was performed after the assessment interview and before the baseline assessment. Patients were informed whether they had been assigned to the intervention or control group immediately after the baseline measurement. The psychologist was assigned the participant they had assessed, provided the participant was randomized to the intervention group. Block randomization was applied, with groups of 10 eligible adults randomly assigned with a 1:1 allocation o either *I AM* or TAU. An independent individual not involved in the study drew slips of paper from a bowl, with each slip representing one of the two possible assignments. After the ten slips of paper had been drawn, they were put back into the bowl and a new draw began. Once selected, participants received a secure message through the national digital healthcare platform 1177, prompting them to provide consent via BankID. The study's self-assessments were administered via Formulärtjänst, a form service that is part of 1177. Baseline, post and 6-month data were collected during 2023–2024, and the 12-month follow-up during 2024–2025. See Fig. 1 for the CONSORT flow chart of the study.

**Fig. 1:**
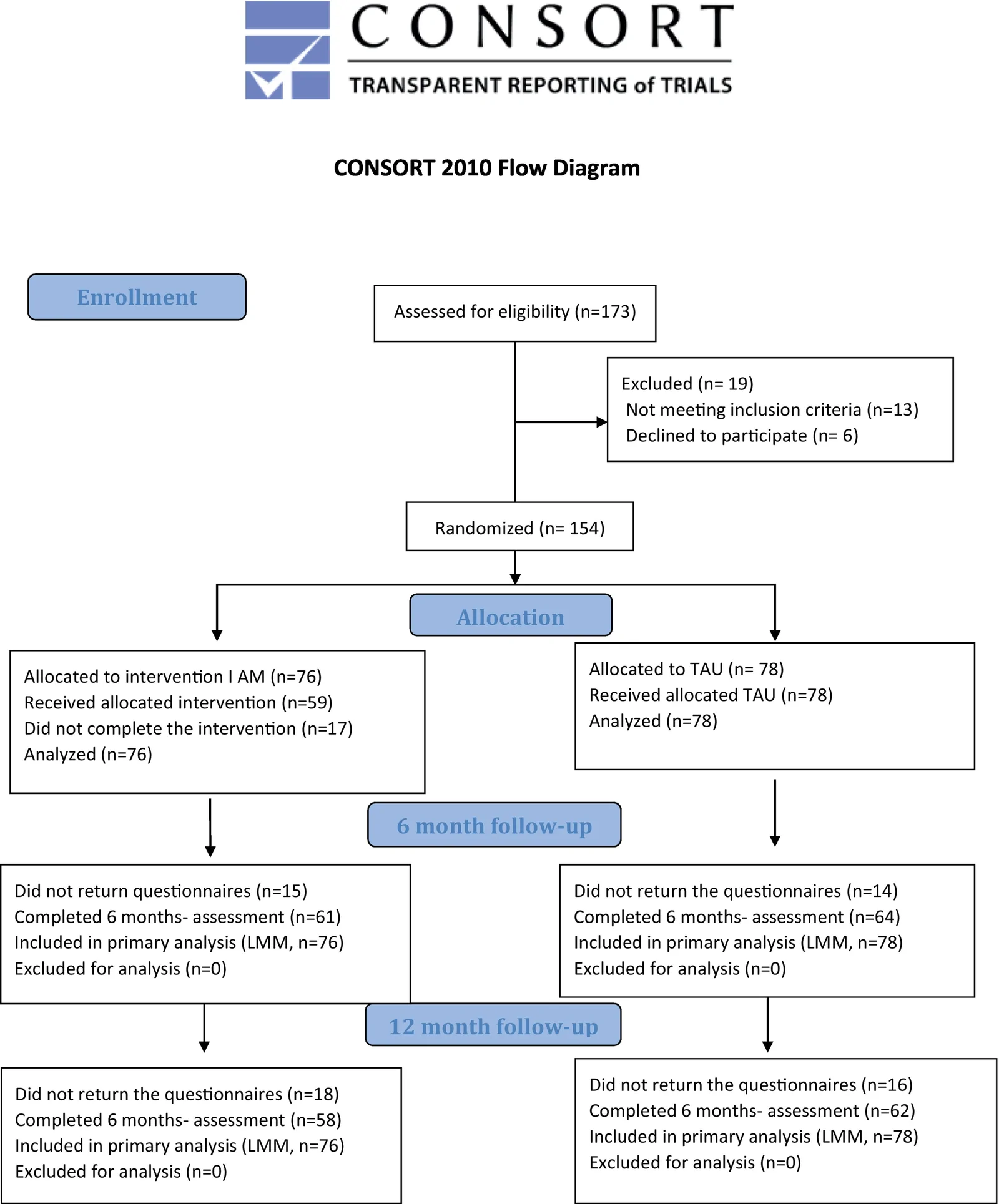
CONSORT flow chart of the study.

### 2.3 Intervention condition: *I AM* (internet approach to migraine)

*I AM* is an acceptance-based CBT intervention informed by principles from ACT, mindfulness, compassion-focused approaches, and contemporary pain psychology. The intervention is based on a biopsychosocial approach and is developed as an add-on to medical treatment. *I AM* complements medical treatment by focusing on functionally managing migraine pain during attacks in accordance with “the golden standard” for medical treatment, which entails pain relief within two hours (Diener et al., 2020). The aim of Acceptance and Commitment Therapy (ACT) is to achieve psychological flexibility. In a migraine context, it may be beneficial to confront the migraine pain and accept the situation at an early stage when the migraine pain is mild during the attack, with sufficient acute medication as prescribed and rest as an act of acceptance and self-compassion (Hayes et al., 2006; Persson et al., 2026). When an individual develops a chronic condition, an adaptation to the new situation begins. The treatment content addresses challenges and risk factors for progression to chronic migraine, such as under- and overuse of acute medication, and offers a proactive approach to improve quality of life content (see Table 1). The theoretical foundation and treatment model of *I AM* have been described in detail in a previous pilot randomized controlled trial (Persson et al., 2024).

**Table 1:** Description of each module titles, contents and targets.

Module	Title	Contents
1	Introduction and goal formulation	Contains information about migraines and pain, as well as a description of the working model used by the treatment program. Introduction of mindfulness practices and goal formulation based on identified values are also included. Physical activity is introduced and gradually increased in duration and intensity for each module.
2	How do I deal with a migraine attack?	Mapping of migraine attacks, using an Educational Migraine Diary with questions such as: What do my migraine attacks look like? The location, character, and intensity of the pain. How do I cope with the attacks? Continued mindfulness exercises.
3	Experience of pain and pain management	Preventive and acute pain management strategies are described and practiced. Physiotherapist-led mobility training.
4	The brain's interpretation of pain	What do I experience during a migraine attack? Mapping experiences with a functional analysis. Strategies for handling thoughts, feelings, and behaviors related to these contribute to making me feel worse.
5	Strategies for dealing with uncertainty	Strategies for managing worries and anxiety related to migraines.
6	Take care of yourself	Strategies for dealing with self-criticism and exercises for showing care to oneself. Description of the self-compassion model.
7	Becoming aware and rethinking fears and avoidances	Evaluating behaviors with guiding questions such as “does fear of new migraine attacks control my life?” Are there things that I do too much of? Or is there something I need to do less of? Psychological exercises such as behavior experiments and exposure.
8	Chronic migraine and the body's pain system	Psychoeducation about the pain system in chronic migraine. Explanation of depression, anxiety, and sleep issues that may accompany the condition. Guidance on communicating about the illness with others.
9	Fear of the consequences of medical treatment	The topic of psychoeducation regarding the importance of taking medication at the appropriate time is being investigated. Questions that are being considered include, “Do you refrain from taking emergency medication as directed during a migraine attack because of concerns about addiction, side effects, fear of not being able to function fully, or due to internal rules or norms? It is important to weigh the pros and cons of this approach and consider if a change in mindset is necessary.”
10	Expanding and sustaining new learning	Maintenance of behavior change and continued training in exercises and strategies. Develop a maintenance plan to manage the disease in the future.

All participants in the intervention group received the medical care offered within healthcare services and were assigned four mandatory modules of the *I AM* program, along with optional modules chosen during the assessment interview. The mandatory modules (module 1,2,3,10) provided information about migraine and pain, pain relief, mindfulness exercises, aerobic training, and physiotherapist-led movement exercises, intended as support for individuals with less severe migraine symptoms. The optional modules (module 4–9) offered more targeted treatment for specific issues such as pain management, experiences during a migraine attack, strategies for handling anxiety, uncertainty, and rumination about causes related to migraine, shame and self-care, finding a balance between too much and too little activity, behavioral experiments, and gradual exposure to avoidance behaviors, such as aerobic training. Additional information about chronic migraine, common fears related to medication, and a maintenance plan were also included, see Table 1.

Based on feedback from the pilot study (Persson et al., 2024), the intervention was customized for the current study to better meet individual needs. This included personalized pacing of treatment modules, tailoring of amount and mode (written messages or telephone contacts) of support from the therapist, and flexible selection of modules. In addition to the mandatory modules, specific modules were recommended based on participants' descriptions of their migraines during the assessment interview, in order to tailor the intervention to individual needs. For example, if a patient reported experiencing numerous distressing thoughts and emotions during attacks that exacerbated their overall suffering, Module 4 was suggested. When patients expressed significant worry or concern about forthcoming migraine attacks, therapists could recommend a personalized approach by adding Module 5, which addresses anxiety and living with uncertainty. If a patient described avoiding a wide range of situations due to fear of triggering a migraine attack, Module 7 was recommended. Participants with chronic migraine were generally advised to complete all modules, particularly Module 8, which focuses on living with chronic migraine. For patients who reported insufficient pain relief during attacks—characterized by severe pain lasting several days—Module 9 was suggested. These refinements aimed to enhance accessibility and engagement, address challenges identified in the pilot phase, and promote improved outcomes in migraine management.

*I AM* was delivered through the Swedish national support and treatment platform (SoB). Three licensed psychologists trained in cognitive behavioral therapy (CBT) and internet-based cognitive behavioral therapy (ICBT) were responsible for assessment and treatment. Participants engaged independently in the *I AM* program, completing one module per week or every two weeks, depending on preferences established during the assessment. The psychologist communicated with participants in writing via a messaging function within the treatment platform, where participants could also send messages to their psychologist. The average time to administer a single module was 15 min. Additionally, participants and psychologists had a follow-up phone call halfway through the treatment and another at the end. The psychologist conducting the assessment interview managed and accompanied the participant throughout the treatment. Participants retained access to *I AM* for an additional three months after completing the treatment.

### 2.4 The control condition: TAU

The treatment as usual (TAU) for migraine stated that patients were allowed to continue with regular migraine treatment according to regional guidelines. According to regional medical guidelines, the focus of migraine treatment is to minimize factors that trigger attacks. The first step of pharmacological treatment of migraine is having an effective acute medication, which involves over-the-counter pain relievers, along with recommendations for rest and sleep. If this approach proves to be ineffective, higher doses may be attempted. The next step would be to add the use of triptan drugs. For individuals experiencing three or more migraine attacks per month with unsatisfactory results from acute medication, preventive drugs such as beta blockers or other preventive treatments in use in primary care should be considered. Some individuals had help from neurologists with more advanced preventative treatments such as botulinum toxin or anti-CGRP treatments. The goal of preventative treatment is to reduce the frequency and severity of migraines by 50%. The study protocol did not control or regulate whether the research subject needed adjustments in their treatment during the course of the study this meant that pharmacological treatments could be changed during the study period in both treatment arms. The participants in the control group could undergo the *I AM* treatment after the last measurement of the study.

### 2.5 Measurements

#### 2.5.1 Demographics and baseline variables

Demographic variables were collected during the assessment interview via questionnaires including information on duration of migraine, medications prescribed, symptoms related to migraine and estimated work capacity. A structured interview was conducted using the Primary Care Evaluation of Mental Disorders (PRIME-MD) to assess potential other mental disorders (Tamburrino et al., 2009).

Three validated self-assessment questionnaires were used in a baseline measurement to describe the population; Clinical Outcomes in Routine Evaluation (CORE-10) consists of ten questions that measure mental health such as degree of depression, anxiety and suicidality. Questions are answered on a 5-point Likert scale with responses ranging from 0 (never) to 4 (always). The clinical cut-off for CORE-10 is 11 and the answers give a total score where higher values indicate higher degree of psychological distress *(*Elfström et al., 2013*)*. Central Sensitization Inventory (CSI, Part A) identifies key symptoms associated with central sensitivity syndrome (CSP). Part A consists of 25 central sensitization-related symptoms rated on a Likert scale from 0 to 4. Each question may be answered as follows: Never (0points), Rarely (point), Sometimes (2 points), Often (3points), or Always (4 points). Following is a breakdown of score ranges and the intensity of CSP they represent. Subclinical: 0 to 29 Mild: 30 to 39 Moderate: 40 to 49 Severe: 50 to 59 Extreme: 60 to 100. The validated cut-off level of ≥30 was used to distinguish between participants with and without central sensitization (Mayer et al., 2012; Neblett, 2018; Schuttert et al., 2023) “FAR” physical activity was measured via a scale used as a measure of achieving public health recommendation level of physical activity. Patients answer two activity questions; 1. How many days during the past week did you engage in at least 30 min of total time (at least 10 min at a time) of physical activity that made you feel slightly out of breath and gave a slightly elevated heart rate, equivalent to brisk walking? 2. How many days during the past week did you engage in some form of strenuous physical activity/exercise, for at least 20 min, that made you feel very short of breath and that gave you an elevated heart rate, equivalent to jogging? The FAR questions operationalize Haskell's definition of sufficient physical activity by converting two self-reported components—number of days and minutes per day—into a weighted score in which minutes are weighted more heavily (1.7×). The sum reflects the total dose of physical activity, and a score of <5 indicates an insufficient level according to (Haskell et al., 2007).

#### 2.5.2 Primary outcomes

*Number of migraine days* were assessed using a custom-developed question for this study: “On average, how many migraine days per month have you had during the past three months?”

*Pain intensity* was measured using a visual analog scale (VAS) in response to: “Estimate how painful your headache was during the past three months on a scale from 0 to 10, where 0 = no pain and 10 = worst pain imaginable.” The VAS is a validated instrument with a continuous scale from 0 to 10, allowing for nuanced reporting of perceived pain intensity (Delgado et al., 2018).

*Migraine burden* was measured with the self-assessment questionnaire HIT-6 (Headache Impact Test). The questionnaire assesses the overall burden of headaches by capturing six key dimensions of impact on daily functioning. These include pain intensity, limitations in physical activities, and the extent to which headaches interfere with work, school, or social functioning. Additionally, the HIT-6 addresses cognitive difficulties such as impaired concentration, emotional aspects such as irritability and mood disturbances, as well as the need to rest or lie down due to headache symptoms. The HIT-6 consists of six questions. Each question can be scored from 6 to 13. The total HIT-6 scores range from 36 to 78. A higher score indicates more disability due to headaches. The four categories of headache severity are: little or no impact (49 or less), Some impact (50–55), Considerable impact (56–59), and Severe impact (60–78) (Kosinski et al., 2003). In the present study, the HIT-6 demonstrated acceptable internal consistency at baseline (Cronbach's α = 0.757).

#### 2.5.3 Secondary outcome

*Mental health and well-being* were measured by the Mental Health Continuum Short Form (MHC-SF). The self-assessment questionnaire consists of 14 questions that focus on emotional, psychological and social well-being and are considered to have good psychometric characteristics (Keyes, 2005). Each question can be scored between 0 and 5, meaning that the total score on the scale can vary from 0 to 70 points. Higher scores indicate a higher level of well-being. In the present study, the MHC-SF demonstrated good internal consistency at baseline (Cronbach's α = 0.896).

### 2.6 Statistical analysis

The primary aim of the statistical analysis was to evaluate whether changes in outcomes over time differed between the *I AM* + treatment as usual (TAU) and TAU-only conditions. Linear mixed models (LMMs) were used to analyze the repeated-measures data, with the time × condition interaction representing the primary test of the intervention effect. Separate models were estimated for migraine days, pain intensity, migraine-related disability (HIT-6), and mental health and well-being (MHC-SF).

The models included time (baseline, post-treatment, 6-month follow-up, and 12-month follow-up), treatment condition (*I AM* + TAU vs. TAU), and the time × condition interaction as fixed effects. Baseline was specified as the reference category for time and TAU as the reference category for treatment condition. Accordingly, the time × condition coefficients represent between-condition differences in change from baseline to post-treatment, 6-month follow-up, and 12-month follow-up. Random intercepts were included to account for repeated observations within participants. Age, sex, migraine type (episodic vs. chronic), and baseline physical activity level were included as covariates. These baseline characteristics were included as potentially prognostic variables that could be associated with the study outcomes and thereby improve the precision of the estimated treatment effects, consistent with methodological guidance on covariate adjustment in randomized clinical trials (Bannick et al., 2026).

Analyses followed a modified intention-to-treat approach, including randomized participants with at least one available outcome measurement for the respective outcome. Linear mixed models (LMMs) were used to account for repeated observations within participants and to incorporate all available outcome data under a maximum likelihood framework. Participants with incomplete follow-up assessments could therefore contribute their available observations without requiring complete data across all assessment points or single-value imputation. Participants without any usable outcome data for a given outcome were not included in the corresponding model. The validity of likelihood-based estimates in the presence of incomplete data relies on the assumption that data are missing at random (MAR). Comparisons between participants who completed follow-up assessments and those who did not indicated no statistically significant differences in the examined demographic characteristics or baseline outcome measures. However, these comparisons do not establish that the MAR assumption holds. Comparisons between participants who completed follow-up assessments and those who did not indicated no statistically significant differences in the examined demographic characteristics or baseline outcome measures.

Estimated marginal means (EMMs) were obtained from the models to describe model-estimated outcome levels by treatment condition and assessment point. The primary interpretation of treatment effects was based on the time × condition interaction terms rather than on post hoc within-condition or single-time-point comparisons. No formal adjustment for multiple testing was applied, and secondary and exploratory analyses were therefore interpreted cautiously. A secondary exploratory categorical analysis examined the association between treatment condition and migraine-frequency category (<15 vs. ≥15 migraine days per month) at the 6-month follow-up using Pearson's chi-square test. Fisher's exact test was performed as a sensitivity analysis. This analysis was considered supplementary and was not used as the primary test of intervention efficacy. Statistical analyses were conducted using SPSS, version 29. Statistical significance was set at a two-sided *p* < .05.

#### 2.6.1 Power analysis

An a priori sample size calculation was conducted using G*Power (version 3.1), based on findings from the initial pilot study. The calculation was based on mental health and well-being, measured using the Mental Health Continuum–Short Form (MHC-SF), for which an effect size of 0.46 was observed at post-treatment in the pilot study. Assuming a significance level of α = 0.05 and statistical power of 0.80, the estimated required sample size was 120 participants (60 per condition). As the calculation was based on MHC-SF data from the pilot study, a corresponding sample size calculation for monthly migraine days was not available. Recognizing that a larger sample might be required to detect effects on migraine frequency and other outcomes, the original recruitment target was set at 200 participants (100 per condition). The final randomized sample comprised 154 participants.

## 3 Results

The main results are presented below, while more detailed findings are provided in Supplement 2: Results.

### 3.1 Participants

A total of 173 people were assessed in the study. Nineteen people were excluded because they did not meet the study criteria. After the assessment, 154 participants were randomized into either the intervention group (*n* = 76), *(I AM* as add on to medical treatment) or the control group (*n* = 78) (medical TAU alone). The participants ranged in age from 19 to 86 years, with a mean age of 45.8 years (SD = 13.2), and the majority were women (88%). Sixty-two percent (62%) had a diagnosis of episodic migraine, while 38% fulfilled the criteria for the diagnosis chronic migraine. The participants had been living with migraines for an average of 26 years (SD = 13.8), with durations ranging from 1.5 to 55 years. Over the past three months, they reported an average of 10 migraine days per month. The pain intensity was estimated at an average of 6.40 on a 10-point scale. Participants estimated a moderate severity of central sensitization (measured with CSI) (Mayer et al., 2012) and the headache had a severe impact on participants' lives (measured with HIT-6) (Kosinski et al., 2003). Ninety-seven percent (97%) of all participants in both groups used acute medicine and 64% used preventive medicine. Fourteen percent (14%) were judged to have comorbidity of psychological symptoms (measured with PRIME-MD). Seventy-six percent (76%) of all participants worked full-time. Participants own subjective work capacity due to migraine disease was estimated at 74%. The participants reported low levels of general psychological distress (measured with Core–10) (Haskell et al., 2007).

### 3.2 Attrition analysis

Eighty percent (*n* = 125) of all randomized participants took part in the 6-month follow-up assessment and 78% (*n* = 120) completed the 12-month follow-up assessment. Participants who completed all assessments did not differ significantly from those who dropped out in terms of demographic or clinical characteristics. To evaluate the validity of the Missing at Random (MAR) assumption applied in the linear mixed model analyses, a comparative analysis was conducted between study completers and dropouts. No statistically significant differences were found between the two groups (*I AM* and TAU) regarding demographic variables (age, sex, migraine type) or baseline clinical outcomes, including number of migraine days, pain intensity, headache-related disability, or Mental health and wellbeing. See supplementary material, Table 1 for demographic characteristic 12 months follow-up.

### 3.3 Treatment preferences, adherence, and follow-up participation

Among participants, 41% (*n* = 29) preferred to complete one module per week, while 59% (*n* = 42) opted for one module every other week (see Table 3). In total, 78% (*n* = 59) completed all of the modules they had selected, in addition to the four mandatory modules. The remaining 22% (*n* = 17) completed, on average, 43% of their chosen modules. Overall, the 76 individuals in the *I AM* group completed 88% of the treatment modules they had selected. On a group level, participants completed an average of 7.4 modules.

**Table 3:** Participants choices in treatment (*I AM*, *N* = 76).

Choices		Frequencies n (%)
Voluntary modules in *I AM*	Module 5	n=61 (81)
	Module 6	n= 60(80)
	Module 9	n= 60(80)
	Module 4	n=56 (74)
	Module 7	n=54 (72)
	Module 8	n= 48 (63)
Treatment rate	one module per week	*n* = 29 (41)
Frequencies (%)	one module every other week	*n*= 42 (59)

### 3.4 Migraine days

The estimated mean number of monthly migraine days in the *I AM* group was 14.2 (SE = 0.80) at baseline, 14.6 (SE = 1.0) at post-treatment, 11.8 (SE = 1.0) at the 6-month follow-up, and 11.7 (SE = 1.1) at the 12-month follow-up. Corresponding estimates in the TAU group were 15.1 (SE = 0.8), 13.7 (SE = 1.0), 13.6 (SE = 1.0), and 13.7 (SE = 1.1), respectively.

Linear mixed model analyses, with baseline as the reference timepoint, showed no statistically significant time × condition interactions for migraine days. The POST × iCBT interaction was non-significant (β = 1.767, 95% CI [−0.842, 4.376], *p* = .183). Similarly, the 6-month × iCBT interaction was non-significant (β = −0.878, 95% CI [−3.497, 1.740], *p* = .509), as was the 12-month × iCBT interaction (β = −1.129, 95% CI [−4.090, 1.832], *p* = .453). Thus, there was no statistically reliable evidence that changes in migraine frequency from baseline to post-treatment, 6 months, or 12 months differed between the *I AM* and TAU groups. Consistent with the interaction analyses, between-group comparisons at post-treatment, 6 months, and 12 months were non-significant (all ps = 0.13–0.45). The between-group effect size for migraine days was 0.26 (95% CI [−2.34, 1.95]). Estimated marginal means and associated *p*-values are presented in Table 4, and detailed interaction effects are provided in Table 2. A secondary categorical analysis examined the proportion of participants reporting ≥15 migraine days per month at the 6-month follow-up. This frequency threshold was used as a clinically interpretable secondary categorization and should not be considered equivalent to fulfilling the full diagnostic criteria for chronic migraine. At the 6-month follow-up, 14.8% (*n* = 9) of participants in the *I AM* group and 29.7% (*n* = 19) in the TAU group reported ≥15 migraine days per month. The association between treatment condition and migraine-frequency category at 6 months was statistically significant, χ^2^(1, *N* = 125) = 4.01, *p* = .045 (likelihood ratio: χ^2^ = 4.09, *p* = .043; one-sided Fisher's exact test, *p* = .036). Given that this was a secondary categorical analysis and that the primary continuous analysis of migraine days showed no statistically significant time × condition effects at any follow-up assessment, this finding should be interpreted cautiously and is not considered evidence of an overall intervention effect on migraine frequency. See Table 4, Table 5.

**Table 4:** Adjusted means (SE) and *p*-values from LMM.

Variables	I AM *n* = 76							TAU *n* = 78							Between, *p*-value		
	Pre M(SE)	Post M(SE)	6 m M(SE)	12 m M(SE)	Within group p-value			Pre M(SE)	Post M(SE)	6 m M(SE)	12 m M(SE)	Within group p-value			Post	6 m	12 m
					Pre-post	Pre – 6 m	Pre – 12 m					Pre-post	Pre – 6 m	Pre – 12 m			
Migraine days	14.2(0.80)	14.6(1.0)	11.8 (1.0)	11.7 (1.1)	0.73	**0.012**⁎	**0.019**⁎	15.1 (0.8)	13.7 (1.0)	13.6 (1.0)	13.7 (1.1)	0.12	0.10	0.18	0.45	0.13	0.14
Pain intensity	6.2 (0.26)	5.5 (0.28)	5.4 (0.31)	5.0 (0.30)	**0.010**⁎	**0.011**⁎	**< 0.001**⁎	6.5 (0.26)	6.3 (0.27)	6.0 (0.30)	5.7 (0.29)	0.51	0.094	**0.003**⁎	**0.004**⁎	0.075	**0.043**⁎
HIT-6	63.9 (0.84)	61.2 (1.1)	58.8 (1.3)	57.5 (1.1)	**0.004**⁎	**< 0.001**⁎	**< 0.001**⁎	64.4 (0.83)	62.7 (1.0)	61.3 (1.26)	61.4 (1.0)	0.054	**0.008**⁎	**0.001**⁎	0.21	0.12	**0.001**⁎
MHC -SF	41.0 (2.0)	44.3 (2.1)	44.1 (2.4)	44.7 (2.1)	0.093	0.16	0.061	41.5 (1.9)	39.3 (2.1)	42.1 (2.3)	39.6 (2.0)	0.26	0.79	0.33	**0.027**⁎	0.44	**0.023**⁎

⁎ *P* < .05 Migraine days: How many migraine days per month have you had on average in the last 3 months? Pain Intensity: Estimate on a scale of 1–10 how painful the headache was. 0 = no pain and 10 = worst pain imaginable. HIT-6 = Headache Impact Test-6, MHC SF = Mental Health Continuum Short Form.

**Table 2:** Demographics and baseline characteristic for the study population (*n* = 154).

Variable		Intervention (I AM), *N* = 76	Control (TAU), *N* = 78	Total *N* = 154
Age				
Mean (sd)		45,5 (13.7)	46.2 (12.7)	45.8(13.2)
Gender	Woman	67 (88.2)	69 (88.5)	136 (88)
	Man	9 (11.8)	9 (11.5)	18 (12)
Frequencies (%)				
Diagnosis	Episodic migraine	46 (61.3)	47 (61.8)	93 (61.6)
	Chronic migraine	29 (38.7)	29 (38.2)	58 (38.4)
Frequency (%)				
Frequency migraine days of the three last month Mean (sd)		10.0 (6.3)	10.2 (6.3)	10.1 (6.3)
Pain intensity Mean (sd)		6.2 (1.6)	6.5 (1.7)	6.4 (1.6)
Years lived with migraines Mean (sd)		25.8 (14.2)	25.9 (13.6)	25.9(13.8)
Occupation Frequencies (%)	Employed, full-time	55 (72.4)	62 (80.5)	117 (76.5)
	Unemployed	3 (3.9)	0 (0)	3 (2.0)
	Student	5 (6.6)	4 (5.2)	9 (5.9)
	Sick-leave	2 (2.6)	2 (2.6)	4 (2.6)
	Sickness pensioner, Long-term sick leave	5 (6.6)	3 (3.9)	8 (5.2)
	Parental leave	0 (0)	1 (1.3)	1 (0.7)
	Pensioner	6 (7.9)	5 (6.5)	11 (7.2)
Perceived workability: In the best of worlds, what percentage of full-time do you feel is appropriate to work for you to feel as good as possible in your illness? Mean (sd)		74.3 (21.5)	74.6 (19.6)	74.5(20,5)
Emergency medicine	Yes, prescription coated	*N* = 63 (85.1)	*N* = 65 (85.5)	128 (85.3)
	Yes, non-prescription	*N* = 9 (12.2)	N = 9 (11.8)	18 (12.0)
	No	*N* = 2 (2.7)	N = 2 (2.6)	4 (2.7)
Frequency (%)				
Preventive medicine	Yes	49 (64.5)	48 (64)	97 (64.2)
	No	27 (35.5)	27 (36)	54 (35.8)
Frequency (%)				
Core − 10 Mean (sd)		10.2 (6.5)	9.6 (5.9)	9.9 (6.2)
Comorbidity of psychiatric symptoms (PRIME-MD)	Yes	9 (12.3)	12 (16.2)	21 (14.3)
	No	41 (56.2)	41 (55.4)	82 (55.8)
	Mild symptoms	23 (31.5)	21 (28.4)	44 (29.9)
Frequency (%)				
Csi total pre Mean (sd)		44.4 (12.7)	44.0 (13.3)	44,2(13.0)
Physical activity question 1 Mean (sd)		2.7 (2.0)	3.5 (1.9)	3.1 (2.0)
Physical activity question 2 Mean (sd)		1.2 (1.5)	1.4 (1.6)	1.3 (1.5)

**Table 5:** Linear Mixed Model Analyses to Estimate Interaction Effects Across Outcome Measures.

Variables and [Timepoint] × [Group]	Beta Estimate Variables and [Timepoint] × [Group]	p-value	95% CI Lower	95% CI Upper
Migraine days				
12 Months × iCBT	−1.129	0.453	−4.090	1.832
6 Months × iCBT	−0.878	0.509	−3.497	1.740
POST × iCBT	1.767	0.183	−0.842	4.376
PRE × iCBT	Reference	Reference		
12 Months × Control	Reference	Reference		
6 Months × Control	Reference	Reference		
POST × Control	Reference	Reference		
PRE × Control	Reference	Reference		

Pain intensity				
12 Months × iCBT	−0.303	0.433	−1.063	0.457
6 Months × iCBT	−0.262	0.515	−1.055	0.530
POST × iCBT	−0.511	0.159	−1.225	0.202
PRE × iCBT	Reference	Reference		
12 Months × Control	Reference	Reference		
6 Months × Control	Reference	Reference		
POST × Control	Reference	Reference		
PRE × Control	Reference	Reference		

HIT-6				
12 Months × iCBT	**−3.406**	**0.010**	**−5.970**	**−0.841**
6 Months × iCBT	−1.986	0.236	−5.279	1.308
POST × iCBT	−0.948	0.461	−3.480	1.584
PRE × iCBT	Reference	Reference		
12 Months × Control	Reference	Reference		
6 Months × Control	Reference	Reference		
POST × Control	Reference	Reference		
PRE × Control	Reference	Reference		

MHC -SF				
12 Months × iCBT	**5.563**	**0.043**	**0.176**	**10.950**
6 Months × iCBT	2.539	0.410	−3.525	8.603
POST × iCBT	**5.472**	**0.047**	**0.071**	**10.873**
PRE × iCBT	Reference	Reference		
12 Months × Control	Reference	Reference		
6 Months × Control	Reference	Reference		
POST × Control	Reference	Reference		
PRE × Control	Reference	Reference		

* Control group and Baseline (PRE) were specified as reference categories in the linear mixed model analyses.

***ICBT*** ***=*** *Internet*-*delivered cognitive behavioral therapy, HIT-6* *= Headache Impact Test,MHC-SF = Mental Health Continuum Short Form.*

### 3.5 Pain intensity

Estimated pain intensity in the *I AM* group decreased from 6.2 (SE = 0.26) at baseline to 5.5 (SE = 0.28) post-treatment, 5.4 (SE = 0.31) at 6 months, and 5.0 (SE = 0.30) at 12 months. Corresponding estimates in the TAU group were 6.5 (SE = 0.26), 6.3 (SE = 0.27), 6.0 (SE = 0.30), and 5.7 (SE = 0.29), respectively.

Linear mixed model analyses, with baseline as the reference timepoint, showed no statistically significant time × condition interactions for pain intensity. The POST × iCBT interaction was non-significant (β = −0.511, 95% CI [−1.225, 0.202], *p* = .159). The 6-month × iCBT interaction was also non-significant (β = −0.262, 95% CI [−1.055, 0.530], *p* = .515), as was the 12-month × iCBT interaction (β = −0.303, 95% CI [−1.063, 0.457], *p* = .433). Thus, there was no statistically reliable evidence that changes in pain intensity from baseline to post-treatment, 6 months, or 12 months differed between the *I AM* and TAU groups.

Between-group comparisons at individual assessment points were significant at post-treatment (*p* = .004) and 12 months (*p* = .043), with a non-significant difference at 6 months (*p* = .075). However, these individual contrasts should be interpreted cautiously given the absence of significant time × condition interactions. The between-group effect size for pain intensity was 0.28 (95% CI [−0.79, 0.15]). Detailed interaction effects are provided in Table 2.

### 3.6 Impact of headaches on daily life, severity, and functional burden (HIT-6)

Estimated HIT-6 scores in the *I AM* group decreased from 63.9 (SE = 0.84) at baseline to 61.2 (SE = 1.1) post-treatment, 58.8 (SE = 1.3) at 6 months, and 57.5 (SE = 1.1) at 12 months. In the TAU group, corresponding scores were 64.4 (SE = 0.83), 62.7 (SE = 1.0), 61.3 (SE = 1.26), and 61.4 (SE = 1.0), respectively.

Linear mixed model analyses, with baseline as the reference timepoint, showed that the time × condition interaction for HIT-6 was not statistically significant at post-treatment (β = −0.948, 95% CI [−3.480, 1.584], *p* = .461) or at the 6-month follow-up (β = −1.986, 95% CI [−5.279, 1.308], *p* = .236). However, a statistically significant interaction was observed at the 12-month follow-up (β = −3.406, 95% CI [−5.970, −0.841], *p* = .010). This indicates that the reduction in migraine-related disability from baseline to 12 months was significantly greater in the *I AM* group than in the TAU group. Thus, there was no statistically reliable evidence of a differential treatment effect on migraine-related disability at post-treatment or 6 months, whereas a significant between-condition difference in change from baseline emerged at the 12-month follow-up. The between-group effect size for migraine burden was 0.41 (95% CI [−2.26, 1.18]). Detailed interaction effects are provided in Table 2.

### 3.7 Mental health and well-being (MHC-SF)

Estimated MHC-SF scores in the *I AM* group were 41.0 (SE = 2.0) at baseline, 44.3 (SE = 2.1) post-treatment, 44.1 (SE = 2.4) at 6 months, and 44.7 (SE = 2.1) at 12 months. Corresponding scores in the TAU group were 41.5 (SE = 1.9), 39.3 (SE = 2.1), 42.1 (SE = 2.3), and 39.6 (SE = 2.0), respectively. Linear mixed model analyses, with baseline as the reference timepoint, showed statistically significant time × condition interactions for mental health and well-being at post-treatment and at the 12-month follow-up. At post-treatment, the POST × iCBT interaction was significant (β = 5.472, 95% CI [0.071, 10.873], *p* = .047), indicating a greater improvement in MHC-SF scores from baseline to post-treatment in the *I AM* group compared with the TAU group.

The interaction at the 6-month follow-up was not statistically significant (β = 2.539, 95% CI [−3.525, 8.603], *p* = .410), indicating no statistically reliable difference between conditions in change from baseline to 6 months. At the 12-month follow-up, the time × condition interaction was statistically significant (β = 5.563, 95% CI [0.176, 10.950], *p* = .043), indicating that improvement in mental health and well-being from baseline to 12 months was significantly greater in the *I AM* group than in the TAU group.

Taken together, these findings indicate differential improvements in mental health and well-being favoring *I AM* + TAU at post-treatment and 12 months, but not at the 6-month follow-up. The between-group effect size for mental health and well-being was 0.29 (95% CI [−3.08, 3.85]). Detailed interaction effects are provided in Table 2.

## 4 Discussion

The present randomized controlled trial evaluated an individually tailored, therapist-guided, acceptance-based Internet-delivered CBT intervention (*I AM*) as an adjunct to treatment as usual for individuals with frequent episodic and chronic migraine in primary care. Overall, the pattern of findings suggests that the effects of *I AM* were more evident in migraine-related disability and mental health and well-being than in migraine frequency or pain intensity. Compared with TAU alone, *I AM* + TAU was associated with a greater reduction in migraine-related disability at the 12-month follow-up and greater improvements in mental health and well-being at post-treatment and 12 months. In contrast, no statistically significant differential effects were found for migraine frequency or pain intensity. Rather than considering these outcomes separately, this pattern may be particularly informative when interpreted in relation to the theoretical aims of acceptance-based treatment and previous research on ACT for chronic pain and migraine.

The findings regarding disability and mental health are broadly consistent with previous research suggesting that the effects of acceptance-based interventions may be particularly evident in functioning and psychological outcomes. Acceptance, values-based action, and psychological flexibility have been associated with better functioning and psychological outcomes in chronic pain (McCracken and Vowles, 2014), and systematic reviews and meta-analyses have found beneficial effects of ACT on pain-related functioning, pain acceptance, psychological flexibility, quality of life, anxiety, and depression (Trindade et al., 2021; Ma et al., 2023). Importantly, these effects do not necessarily depend on substantial reductions in pain itself. This distinction is particularly relevant to the present findings, as *I AM* was associated with improvements in migraine-related disability and mental health and well-being despite no statistically reliable differential effects on pain intensity or migraine frequency.

The present results are also consistent with the more specific literature on acceptance-based interventions for migraine. In a meta-analysis, Polk and Smitherman (2023) found a significant improvement in migraine-related disability following acceptance- and mindfulness-based interventions, whereas effects on headache frequency and medication use were not statistically significant. This closely resembles the overall pattern observed in the present study. The convergence between these findings may indicate that the principal benefits of acceptance-based treatment for migraine are not necessarily best captured by the number of migraine days alone. Instead, treatment may help individuals reduce the extent to which migraine interferes with daily activities, psychological well-being, and valued areas of life. Such an interpretation is consistent with the theoretical model underlying ACT. The primary aim of ACT is not symptom elimination, but to increase psychological flexibility and support engagement in meaningful activities in the presence of difficult internal experiences or persistent symptoms (Hayes et al., 2011; Ong and Eustis, 2021). From this perspective, a person may experience a similar number of migraine days while becoming less restricted by migraine during and between attacks. The significant reduction in migraine-related disability at 12 months, together with improvements in mental health and well-being, is compatible with this possibility. However, psychological flexibility, acceptance, and values-based action were not examined as mediators in the present analyses. The proposed explanation should therefore be regarded as theoretically plausible rather than as evidence of a demonstrated treatment mechanism.

The absence of a significant effect on migraine frequency should also be considered in the broader context of migraine treatment research. Detecting between-group differences in monthly migraine days is challenging because migraine frequency fluctuates considerably over time and because clinically meaningful improvements may occur in both intervention and comparison conditions. This is particularly relevant in pragmatic trials in which participants continue to receive usual medical care. Even in large pharmacological migraine trials, statistically significant average between-group effects on monthly migraine days can be relatively modest. Meta-analytic evidence for CGRP monoclonal antibodies, for example, has reported placebo-adjusted reductions of approximately 1.4–1.6 monthly migraine days, despite considerably larger pooled samples than in the present trial (Robblee et al., 2024). This illustrates that mean between-group differences in migraine frequency may be difficult to detect even for established migraine-specific preventive treatments.

This comparison is not intended to imply equivalence between psychological and pharmacological treatment effects, as the interventions, mechanisms, study populations, and trial designs differ substantially. Rather, it highlights an important methodological issue in migraine research: average changes in migraine days may provide only one perspective on treatment benefit. A relatively modest average group difference can coexist with substantial improvement among individual patients, while heterogeneity in response may attenuate the group-level effect. The secondary finding that fewer participants in the I AM group met the threshold for chronic migraine at the 6-month follow-up may be relevant from this perspective, but it should remain exploratory because the primary continuous analysis of migraine days did not demonstrate a significant time × condition interaction.

The distinction between migraine frequency and migraine-related functioning may therefore be clinically important. For patients living with frequent or chronic migraine, treatment goals may include not only fewer attacks but also improved ability to work, maintain social relationships, engage in valued activities, and manage the psychological consequences of an unpredictable and disabling condition. The present findings suggest that these broader outcomes may be particularly relevant when evaluating acceptance-based interventions. This is also supported by chronic pain research showing that psychological flexibility and pain acceptance are related to functioning and psychological adjustment and that ACT can improve functional and psychological outcomes even when effects on pain itself are smaller or inconsistent (Ye et al., 2024).Thus, assessment of migraine days alone may underestimate changes that are meaningful to patients.

The improvements in mental health and well-being further support this broader perspective. Individuals with frequent migraine experience a substantial psychological burden, and psychological well-being is clinically relevant independently of changes in headache frequency. The greater improvement in MHC-SF observed in the *I AM* group at post-treatment and 12 months suggests that an acceptance-based intervention may influence dimensions of health that are not captured by conventional headache indices. The lack of a significant differential effect at 6 months, however, indicates that this pattern was not uniform across all assessment points. Consequently, the findings should not be interpreted as demonstrating a continuous or steadily increasing effect over time. Replication with studies designed to examine treatment mechanisms are needed to determine whether the longer-term pattern reflects consolidation of behavioral changes, variability in migraine over time, or other processes.

The delayed differential effect on migraine-related disability is also noteworthy. A significant between-condition difference in HIT-6 was observed at 12 months but not immediately after treatment or at 6 months. One possibility is that behavioral changes promoted by an acceptance-based intervention require time to become integrated into everyday life. Learning to engage in valued activities despite migraine, alter responses to symptoms, and reduce avoidance may conceivably have cumulative effects on functioning. Internet-delivered ACT interventions for chronic pain have previously demonstrated that acceptance-based processes can be targeted through digital treatment formats (Buhrman et al., 2013; Lin et al., 2017; Trindade et al., 2021). Nevertheless, because the present study did not directly test mediation or trajectories of psychological flexibility, the delayed effect should not be interpreted as evidence for such a mechanism. The temporal pattern warrants further investigation.

The individually tailored format of *I AM* may also have contributed to its clinical applicability. Participants could receive treatment adapted to their needs in terms of module selection, pacing, and therapist guidance. Such flexibility may be particularly valuable for migraine, where symptom burden, fatigue, cognitive difficulties, and capacity to engage in treatment can vary considerably over time. Previous research has indicated that individually tailored ICBT can facilitate engagement among patients with heterogeneous or comorbid presentations (Kraepelien et al., 2019; Nye et al., 2023). The present study cannot determine whether tailoring itself improved treatment outcomes, but the combination of relatively high adherence and sustained follow-up participation supports the feasibility of delivering this type of intervention in routine clinical care.

Taken together, the results extend previous findings on acceptance-based treatment by evaluating a therapist-guided digital intervention within routine primary care and with follow-up extending to 12 months. The findings are consistent with previous research suggesting that acceptance-based interventions may have their clearest effects on disability, functioning, and psychological outcomes rather than necessarily producing large changes in the frequency or intensity of pain or migraine. They also emphasize the importance of using multidimensional outcome assessment in migraine trials. For an intervention explicitly designed to increase functioning in the presence of symptoms, outcomes reflecting disability, mental health, and participation may be as important as conventional symptom-frequency measures.

### 4.1 Strengths and limitations

Several strengths increase confidence in the interpretation and clinical relevance of the present findings. First, the randomized controlled design with an active TAU comparison enabled differential changes between conditions to be distinguished from changes occurring over time in both groups. This is particularly important in migraine, given natural fluctuations in headache frequency, regression to the mean, and ongoing medical treatment.

Second, the study achieved its prespecified recruitment target based on the power calculation. The power analysis indicated a target sample of 150 participants, and 154 participants were included in the randomized trial. Thus, the study was successfully powered according to its a priori assumptions. Nevertheless, achieving the planned sample size does not imply that the study was sufficiently powered to detect every possible effect, particularly small between-condition differences across multiple outcomes and follow-up assessments. This distinction is relevant when interpreting the non-significant interaction effects for migraine frequency and pain intensity.

Third, participant retention was high, including at the 6- and 12-month follow-ups. This is an important strength in a longitudinal behavioral intervention study, as attrition over extended follow-up periods can introduce bias and substantially reduce statistical precision. The relatively high response rates therefore strengthen the longitudinal evaluation and enabled examination of whether treatment-related differences were maintained or emerged beyond the immediate post-treatment period.

Fourth, the intervention was evaluated within routine primary care rather than under highly controlled experimental conditions. Participants continued to receive usual medical treatment, making the study more representative of how a psychological intervention would be used in clinical practice: as a complement to rather than a replacement for medical migraine management. This pragmatic context increases the ecological and clinical relevance of the findings, although it simultaneously reduces experimental control.

Several limitations should nevertheless be considered. Participants in both groups continued to receive TAU, including potential changes in pharmacological and other migraine treatment during follow-up. Improvements occurring in both groups can therefore reflect medical treatment, natural variation, regression to the mean, or other factors unrelated to I AM. For this reason, the time × condition interactions provide the most appropriate basis for conclusions regarding differential intervention effects.

Migraine frequency and the other primary outcomes were based on self-report and may therefore be affected by recall and measurement error. Migraine frequency also varies substantially within individuals over time, making stable between-group effects difficult to demonstrate in samples of this size. Moreover, although the planned sample size was achieved, the trial may still have had limited statistical power for small effects, particularly across several follow-up points. The absence of significant interaction effects for migraine frequency and pain intensity should consequently be interpreted as an absence of statistically reliable evidence for differential effects in this trial rather than definitive evidence that the intervention cannot influence these outcomes.

Multiple outcomes and assessment points were examined, which increases the risk of Type I error. Greater weight should therefore be given to the prespecified time × condition analyses than to within-group changes or isolated comparisons at individual assessment points. The secondary analysis indicating a lower proportion of participants meeting the threshold for chronic migraine at 6 months should similarly be interpreted as exploratory.

Finally, the sample consisted predominantly of women. Although this is consistent with the higher prevalence of migraine among women, it may restrict generalizability to men and to populations that differ from the present primary care sample. Future studies should also examine potential mechanisms of change, particularly psychological flexibility, pain acceptance, values-based action, and avoidance, to determine whether changes in these processes account for improvements in disability and mental health independently of migraine frequency.

## 5 Conclusion

The present findings suggest that adding individually tailored, therapist-guided, acceptance-based ICBT to usual migraine care may have benefits that are more apparent in migraine-related disability and psychological well-being than in migraine frequency or pain intensity. This pattern is consistent with previous research on acceptance-based interventions for chronic pain and migraine, in which improvements in functioning and disability have been observed even when effects on symptom frequency are limited.

These findings therefore underscore the importance of evaluating migraine treatment using outcomes that capture not only how often migraine occurs, but also how substantially it restricts everyday life and psychological well-being. *I AM* was evaluated under routine primary care conditions, achieved its prespecified recruitment target, and retained a high proportion of participants through long-term follow-up. Further adequately powered studies should replicate the observed effects and directly examine whether changes in psychological flexibility and related acceptance-based processes mediate improvements in migraine-related functioning and mental health.

## Authors' contributions

M.P. and S·W. planned and designed the study, developed the theoretical framework underlying the intervention, and develop the *I AM* treatment program. S.W. and N.A. assisted M.P. with the statistical analyses. M.P. and S.W. analyzed and interpreted the findings, contextualized the results in relation to the theoretical framework and previous research, and drafted the manuscript. S.W, A.S. and B.D. supervised M.P. throughout the research project and contributed to the interpretation of findings, review and revision of the manuscript. All authors reviewed the manuscript and approved the final version.

## Ethics declaration

The study was approved by the Swedish Regional Ethical Review Board in Gothenburg (Dnr 2022-03880-01, Dnr 2023-03427-02). All participants provided electronic informed consent prior to participation, in accordance with the Declaration of Helsinki (Association, 2025).

## Declaration of Generative AI and AI-assisted technologies in the writing process

In working on this manuscript, the authors used ChatGPT-3.5, an AI language model developed by OpenAI, which helped improve the language and fluency of the text. The authors take full responsibility for the content of the manuscript.

## Declaration of competing interest

No potential conflict of interest was reported by the author(s). The research received grants from 10.13039/501100009750Kamprad Family Foundation [20223182]. Regional and Local Research and Development Council of Södra Älvsborg VGFOUREG-1011475 and VGFOUSA-P-1015615.

## Data Availability

Availability of data and materials from this study is available from the corresponding author upon reasonable request.

## Appendix A Supplementary data

Table 1 Demographic characteristic 12 months follow-up.
