# Supplementary material for: Internet-delivered acceptance-based CBT for frequent migraine in routine care as add-on to medical treatment 12-month results: A randomized controlled trial
Source: Internet Interv. 2026 Nov 5;46:101022. doi: 10.1016/j.invent.2026.101022 (PMC13770601; doi:10.1016/j.invent.2026.101022)
Supplement: Supplementary file 1 [file mmc1.docx]

| **Variabels** | | **Total Baseline**  **N=154** | **1 year follow-up** | |
| --- | --- | --- | --- | --- |
|  |  |  | **participants**  **N=120** | **Drops out**  **N=34** |
| **Age**  Mean (sd) |  | 45.84(13.21) | 46.83 (13.1) | 42.32 (13.2) |
| **Gender**  n (%) | Woman | 136 (88) | 105 (87.5) | 31 (91.2) |
|  | Man | 18 (12) | 15 (12.5) | 3 (8.8) |
|  |  |  |  |  |
| **Diagnosis**  Frequency (%) | Episodic migraine (EM) | 93 (61.6) | 72 (60.0) | 21 (65.6) |
|  | Chronic migraine (CM) | 58 (38.4) | 47 (39.2) | 11 (34.4) |
| **Migraine days of the month**  Mean (sd) |  | 10.09 (6.28) | 9.73 (6.3) | 11.47 (5.9) |
| **Pain intensity**  Mean (sd) |  | 6.40(1.65) | 6.35 (1.64) | 6.56 (1.67) |
| **Years lived with migraines**  Mean (sd) |  | 25.87(13.84) | 26.36 (14.0) | 24.06 (13.2) |
| **Occupation**  Frequencies (%) | Employed | 117 (76.5) | 87 (72.5) | 30 (90.9) |
|  | Unemployed | 3 (2.0) | 3 (2.5) | 0 |
|  | Student | 9 (5.9) | 8 (6.7) | 1 (3.0) |
|  | Sick-leave | 4 (2.6) | 4 (3.3) | 0 |
|  | Sickness pensioner, Long-term sick leave | 8 (5.2) | 8 (6.7) | 0 |
|  | Parental leave | 1 (0.7) | 0 | 1 (3.0) |
|  | Pensioner | 11(7.2) | 10 (8.3) | 1 (3.0) |
| **Perceived workability % Full time**  Mean (sd) |  | 74.5 (2.5) | 73.6 (20.6) | 77.4 (20.2) |
| **Emergency medication use**  Frequency (%) | Yes, prescription coated | 128 (85.3) | 101 (86.3) | 27 (81.8) |
|  | Yes, non-prescription | 18 (12.0) | 13 (11.1) | 5 (15.2) |
|  | No | 4 (2.7 | 3 (2.6) | 1 (3.0) |
| **On Preventive medicine** | Yes | 97 (64.2) | 74 (62.2) | 23 (71.9) |
| Frequency (%) | No | 54 (35.8) | 45 (37.8) | 9 (28.1) |

Table 1 Demographic characteristic 12 months follow-up
